# Post-COVID-19 pandemic changes in pertussis incidence among patients with acute respiratory tract infections in Zhejiang, China

**DOI:** 10.3389/fmicb.2024.1448997

**Published:** 2024-08-09

**Authors:** Huabin Wang, Miao Fu, Wei Chen, Yongjun Ma

**Affiliations:** Department of Clinical Laboratory, Affiliated Jinhua Hospital, Zhejiang University School of Medicine, Jinhua, China

**Keywords:** pertussis, post-COVID-19 pandemic, epidemiology, acute respiratory tract infection, public health

## Abstract

**Background:**

Previous studies have compared the incidence of pertussis before and during the COVID-19 pandemic, finding that public health measures related to COVID-19 contributed to a temporary decline in reported pertussis cases during the pandemic. However, the post-pandemic period has seen a resurgence in respiratory infections, influenced by relaxed health measures and decreased public vigilance. This study investigates the epidemiological dynamics of pertussis among patients with acute respiratory tract infections (ARTI) in Zhejiang Province, China, providing essential reference information for ongoing public health strategies.

**Methods:**

This study analyzed multicenter data from January 2023 to May 2024, involving 8,560 patients with ARTI from three hospitals in Zhejiang Province. Inclusion criteria included patients who presented with cough symptoms and were clinically diagnosed with either acute upper respiratory tract infections (URTI) or acute lower respiratory tract infections (LRTI), and who had undergone at least one *Bordetella pertussis* DNA test. The study analyzed the epidemiological changes of pertussis positivity rates and their associations with time, age, gender, and diagnosis types (URTI and LRTI).

**Results:**

From January 2023 to May 2024, the positivity rate and testing number for pertussis among patients with ARTI generally showed a gradual increasing pattern. In March 2024, the positivity rate reached its peak at 31.58%, followed by a weekly decline. The overall positivity rate was 23.59%, with no significant differences observed between genders. Pertussis incidence was higher in patients with LRTI (24.49%) compared to those with URTI (18.63%, OR = 1.40, 95% CI: 1.20–1.63, *p* < 0.001) and in outpatients (25.32%) compared to inpatients (6.09%, OR = 4.17, 95% CI: 3.07–5.64, *p* < 0.001). According to a generalized additive model analysis, there was a wave-shaped, non-linear relationship between age and pertussis incidence, with a relatively high rate observed in the 5 to 17 age group, peaking at age 10 (33.85%). Additionally, the impact of age, patient type, and diagnosis type on the pertussis infection rate varied across different age groups.

**Conclusion:**

After the COVID-19 pandemic, the positivity rate of pertussis in Zhejiang Province peaked in early 2024 and then showed a declining pattern. Children and adolescents were particularly affected, emphasizing the need for enhanced vaccination and public health interventions in this population.

## 1 Introduction

Whooping cough, caused by *Bordetella pertussis*, is a highly contagious acute respiratory infection that poses a serious threat to public health (Homaira et al., 2023; Nie et al., 2024). Since the introduction of the whole-cell pertussis vaccine in the national immunization program in 1978, the incidence of whooping cough has significantly declined in China (Mei et al., 2024). Starting in 2007, acellular pertussis vaccines began to replace whole-cell vaccines, and by 2013, the DTaP vaccine had completely taken over, maintaining vaccination coverage rates above 99% (Xu et al., 2015; Mei et al., 2024). In China, the DTaP vaccine is administered at the ages of 3 months, 4 months, 5 months, and 18 months. The estimated average duration of protection from the DTaP vaccine is about 3–4 years, and only 10% of children remain protected 8 years after receiving the last dose (McGirr and Fisman, 2015; Domenech de Cellès et al., 2019). Additionally, studies have reported that even in countries or regions with high vaccination coverage, the incidence of pertussis still shows fluctuations and a resurgence trend (Amirthalingam et al., 2013; Auger et al., 2013; Esposito and Principi, 2018; World Health Organization, 2023; Mei et al., 2024), indicating the need for continuous adjustment of immunization strategies to accommodate pathogen variability and changes in population immunity.

Before the COVID-19 pandemic (2017–2019), the incidence of whooping cough was on the rise in the Beijing area of China (Chen et al., 2022). In 2020, due to the pandemic-induced social distancing and other non-pharmaceutical interventions, the number of whooping cough cases significantly dropped to 4,475, just one-sixth of the cases reported in 2019 (Chen et al., 2022). This decrease may reflect the short-term effectiveness of pandemic control measures in reducing the transmission of respiratory infectious diseases (Hu et al., 2021; Huh et al., 2021; Tessier et al., 2022), including whooping cough. However, as these measures gradually relaxed, a resurgence in whooping cough incidence was reported globally. Denmark experienced a significant peak in whooping cough incidence from August 2023 to February 2024, the highest since the onset of the pandemic (Nordholm et al., 2024). Nordholm et al. reported that although the incidence in Denmark was relatively stable before the pandemic, effective social distancing during the pandemic led to reduced rates. Nevertheless, with the relaxation of these measures, cases rapidly increased, particularly among adolescents, highlighting the need for public health strategies to swiftly adapt to the dynamics of the pandemic.

Although current literature reports changes in the incidence of whooping cough in China before and during the COVID-19 pandemic (Li et al., 2024; Sun et al., 2024), data on the epidemiological dynamics of the disease post-pandemic remains very limited. Especially as social activities gradually normalize, understanding the epidemiological dynamics of whooping cough among different age groups and infection types post-pandemic is crucial for devising effective public health strategies. Therefore, this study aims to explore the prevalence of whooping cough among patients with acute respiratory tract infection (ARTI) post-COVID-19 by systematically analyzing case data from three hospitals in Zhejiang Province, in order to provide a scientific basis for future disease control and vaccination strategy adjustments.

## 2 Materials and methods

### 2.1 Study population

This study was based on opportunistic sampling and included patients diagnosed with ARTI from January 2023 to May 2024 at three designated hospitals in Zhejiang Province: the Affiliated Jinhua Hospital, Zhejiang University School of Medicine, Jinhua Maternal and Child Health Care Hospital, and Wucheng District First People’s Hospital. Inclusion criteria included patients who presented with cough symptoms and were clinically diagnosed with either acute upper respiratory tract infections (URTI) or acute lower respiratory tract infections (LRTI), and who had undergone at least one *Bordetella pertussis* DNA test. Exclusion criteria were strictly defined as follows: (1) Patients subjected to multiple *Bordetella pertussis* DNA tests within a 30-day period yielding exclusively negative results were excluded, except for their initial test result. (2) For those testing positive for *Bordetella pertussis* DNA multiple times within a 30-day interval, only the initial positive result was considered. Subsequent tests aimed at monitoring disease progression or regression were excluded. (3) Patients who did not undergo a *Bordetella pertussis* DNA test or whose diagnostic information was incomplete were also excluded. It is pertinent to highlight that from January to December 2023, due to the low incidence of positive pertussis cases in China, *Bordetella pertussis* DNA testing was only conducted in patients highly suspected of having pertussis after other common pathogens had been ruled out, particularly those whose cough symptoms persisted for more than 2 weeks without alleviation. However, given the rapid increase in positive pertussis cases in 2024, the scope of testing was expanded nationally. Nearly all patients with cough symptoms who visited the hospital for ARTI underwent *Bordetella pertussis* DNA testing. This expansion was aimed at enhancing the assessment of the pertussis incidence rate among all patients presenting with cough symptoms indicative of ARTI. According to the inclusion and exclusion criteria, this study enrolled 8,560 patients out of 9,073 from the three hospitals. Of these, 4,183 were female (48.87%), 1,315 had URTI and 7,245 had LRTI, accounting for 15.36 and 84.64%, respectively. Outpatients and inpatients numbered 7,788 (90.88%) and 772 (9.02%) respectively. The study received approval from the Ethics Committee of the Jinyi Medical Group, and Affiliated Jinhua Hospital, Zhejiang University School of Medicine, approval code: (2024) Ethics No. (101).

### 2.2 Data collection

All data for this study were obtained from electronic medical records, including patient age, gender, pertussis test results, diagnostic type [either upper respiratory tract infection (URTI) or lower respiratory tract infection (LRTI)], and patient type (inpatient or outpatient). The nucleic acid testing for *Bordetella pertussis* was conducted using a PCR fluorescence probe-based test kit produced by Sansure Biotech (Changsha, China). This kit had a minimum detection limit of 400 copies/mL, with good intra-assay and inter-assay reproducibility, and a coefficient of variation of Ct values below 5%. In terms of clinical performance validation, compared with traditional bacterial isolation and identification methods, the sensitivity of this test kit reached 100%. Additionally, regarding specificity, the test kit showed no cross-reactivity with 27 types of respiratory pathogens, including *Bordetella parapertussis*, *Bordetella bronchiseptica*, *Bordetella holmesii*, and Respiratory Syncytial Virus, and other similar pathogens. Nasopharyngeal swab samples from each patient were tested, with each reaction system containing 40 microliters of reagent and 10 microliters of sample template. All tests were analyzed using the Thermo Fisher ABI7500 Real-Time PCR System. Notably, all samples from hospitalized patients were collected and tested after admission, within their respective wards.

### 2.3 Statistical analysis

This study utilized SPSS version 26.0 and R statistical software (version 3.6.1) for data processing and analysis. All categorical variables, including gender, age groups, diagnostic type, and patient type, were summarized as frequencies and proportions. The positivity rate for pertussis and its 95% confidence interval (CI) were estimated using the Wilson method. In the initial phase of the analysis, subjects were divided into the following age groups: 0–4 years, 5–10 years, 11–18 years, 19–40 years, and over 40 years. Further, using generalized additive models (GAM), we modeled the non-linear relationship between age and the probability of pertussis infection, incorporating confounders such as gender, diagnostic type, and patient type. By calculating the first derivative of the probability estimates and identifying points where the sign of the derivative changed, we precisely determined the ages corresponding to peaks and troughs in the predicted probability curve. This analysis revealed complex dynamics between age and the probability of pertussis infection, leading us to refine the age groups into four categories: 0–10 years, 11–27 years, 28–41 years, and over 41 years. Logistic regression models were then reapplied to analyze the impact of gender, diagnostic type, and patient type on the pertussis positivity rate across these age groups.

With a significant increase in the pertussis positivity rate in February 2024, we defined this month as the beginning of an upsurge of pertussis in Zhejiang Province. We also employed heat maps to dynamically display the weekly changes in positivity rates among the groups during the upsurge, providing a visual representation of the epidemic patterns. All statistical tests were conducted with a significance level set at *p* < 0.05.

## 3 Results

### 3.1 Temporal changes in pertussis testing and positivity rates

**Figure 1:**
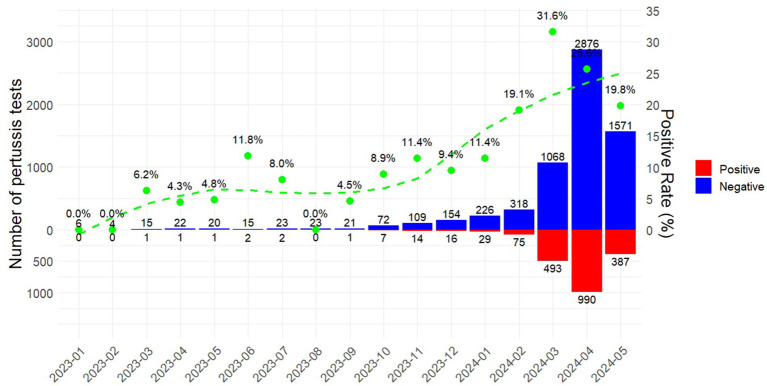
Temporal dynamics of pertussis testing and positivity rates among patients with ARTI, January 2023 to May 2024, in the post-COVID-19 Era.

Figure 1 illustrated the monthly changes in the number of *Bordetella pertussis* DNA tests and positivity rates among ATRI patients from January 2023 to May 2024. During the first 9 months of 2023, both the number of tests and positivity rates remained at relatively low levels. However, beginning in October 2023, both metrics started to gradually increase; particularly in February 2024, the positivity rate sharply rose, reaching a peak of 31.58% in March 2024. Thereafter, there was a slight decline in the positivity rates over the following 2 months.

### 3.2 Characteristics of pertussis detection among patients with ARTI

From January 2023 to May 2024, a total of 8,560 pertussis DNA tests were conducted on patients with ATRI (Table 1). The overall positivity rate was 23.59% (95% CI: 22.69–24.49). Females exhibited a slightly higher positivity rate of 23.95%, compared to 23.24% in males, although this difference was not statistically significant (OR = 0.97, *p* = 0.534). The highest positivity rate was observed in the 5–10 age group at 33.06% (95% CI: 31.58–34.55), significantly surpassing other age groups. In contrast, the positivity rate for the 0–4 age group was the lowest at only 13.24%. The age groups of 19–40 and over 40 years showed positivity rates of 14.66 and 16.34%, respectively. Patients with LRTI registered a positivity rate of 24.49%, notably higher than those with URTI, who had a positivity rate of 18.63% (OR = 1.40, *p* < 0.001). Moreover, outpatients had a positivity rate of 25.32%, significantly higher than the 6.09% observed in inpatients, OR = 4.17, *p* < 0.001.

**Table 1:** The positivity rates of pertussis in patients with ARTI during January 2023 to May 2024.

Group	No. of test	Positive	Positive rate (95 CI)	OR (95 CI)	*p*-value
Overall, *n*	8,560	2,019	23.59 (22.69–24.49)		
**Gender**					
Female	4,183	1,002	23.95 (22.66–25.25)	Reference	—
Male	4,377	1,017	23.24 (21.98–24.49)	0.97 (0.87–1.07)	0.534^a^
**Age, year**					
Age (continuous variable)	8,560	2,019	23.59 (22.69–24.49)	1.00 (0.99–1.01)	0.724^b^
0–4 years	3,256	431	13.24 (12.07–14.40)	Reference	—
5–10 years	3,841	1,270	33.06 (31.58–34.55)	3.00 (2.65–3.39)	<0.001^b^
11–18 years	692	199	28.76 (25.38–32.13)	2.53 (2.08–3.08)	<0.001^b^
19–40 years	416	61	14.66 (11.26–18.06)	1.12 (0.83–1.50)	0.457^b^
Over 40 years	355	58	16.34 (12.49–20.18)	1.50 (1.10–2.03)	0.010^b^
**Diagnosis**					
URTI	1,315	245	18.63 (16.53–20.74)	Reference	—
LRTI	7,245	1,774	24.49 (23.50–25.48)	1.40 (1.20–1.63)	<0.001^c^
**Patient type**					
Inpatients	772	47	6.09 (4.40–7.77)	Reference	—
Outpatients	7,788	1,972	25.32 (24.36–26.29)	4.17 (3.07–5.64)	<0.001^d^

a Adjusted for age, diagnosis and patient type.

b Adjusted for gender, diagnosis and patient type.

c Adjusted for age, gender, and patient type.

d Adjusted for age, gender, and diagnosis type. URTI, upper respiratory tract infections; LRTI, lower respiratory tract infections.

### 3.3 Wave-shaped relationship between age and the pertussis positivity rate

**Figure 2:**
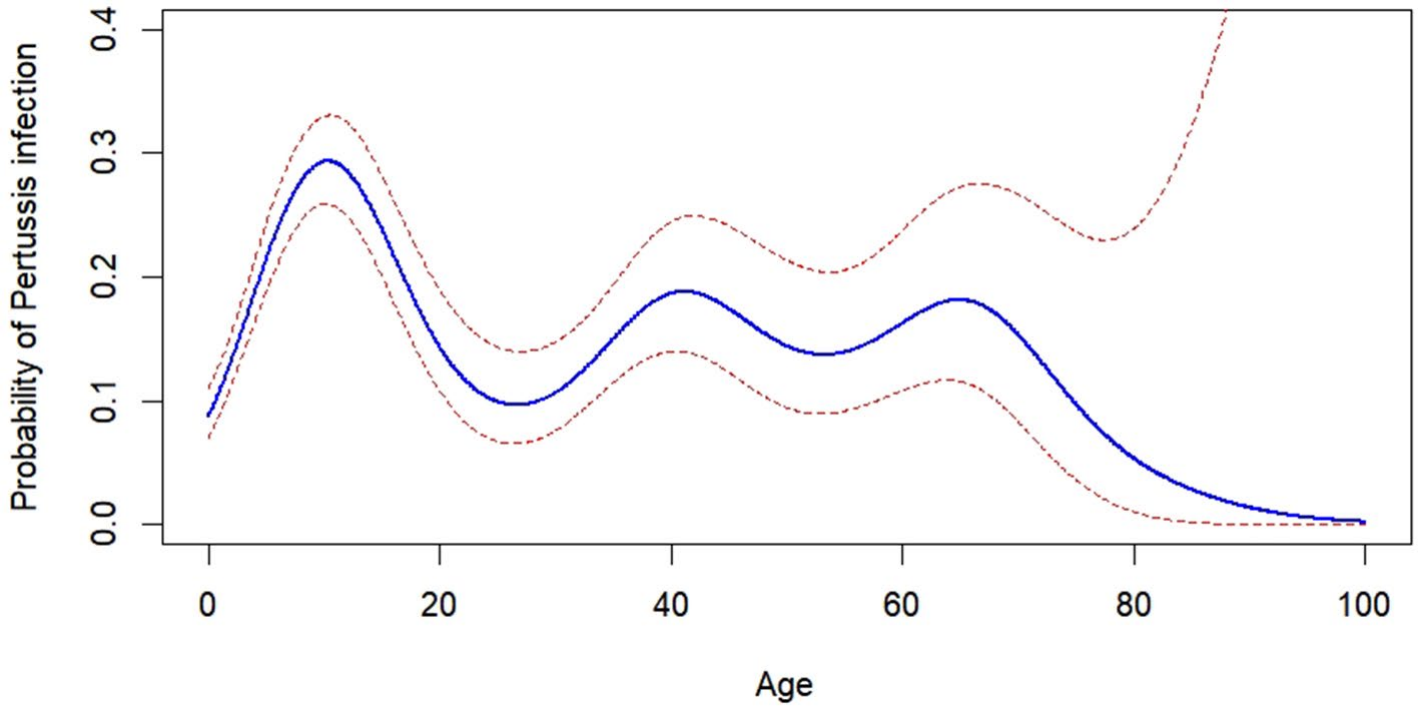
Age-dependent probability of pertussis infection. Analysis using a generalized additive model revealed a non-linear relationship between age and the probability of pertussis infection, incorporating confounders such as gender, diagnosis type, and patient type.

Using a generalized additive model (GAM) and adjusting for confounders such as gender, diagnosis type, and patient type, the analysis revealed a significant wave-shaped non-linear relationship between age and the pertussis positivity rate (*p* < 0.001). Among all participants, ARTI patients aged 5 to 17 showed relatively higher positivity rates, particularly peaking at 33.85% at age 10. Furthermore, the wave-shaped graph identified several key ages—27, 41, 53, and 65—where the pertussis positivity rate displayed significant local maxima or minima (Figure 2).

### 3.4 Analysis of pertussis positivity rates across reclassified age groups

Based on the non-linear, wave-shaped relationship observed between age and pertussis positivity rates, participants were reclassified into different age groups corresponding to the identified peaks and troughs in the analysis. Multivariate logistic regression was then performed for each age group (Table 2), with ORs for each variable adjusted against the three other variables included in the model. In the 0–10 years age group, as age increased, the pertussis positivity rate significantly rose, with each additional year increasing the positivity rate by 17% (OR = 1.17, *p* < 0.001). Additionally, patients with LRTI had a pertussis positivity rate 1.39 times higher than those with URTI (OR = 1.39, *p* < 0.001). Conversely, in the 11–27 years age group, an increase in age was inversely related to the positivity rate of pertussis (OR = 0.92, *p* = 0.005); and although the positivity rate for patients with LRTI was higher than for those with URTI, this difference did not reach statistical significance (OR = 1.43, *p* = 0.101). Moreover, except for the 28–41 years age group, outpatient pertussis positivity rates were significantly higher than those of inpatients across all other age groups. For the age groups above 28 years, the impacts of age, gender, and diagnosis type on the pertussis positivity rate showed no statistical significance.

**Table 2:** Multivariate logistic regression analysis of pertussis positivity rates by age group.

Age, year	Coeff.	Std. Err.	OR (95 CI)	*p*-value^a^
**0–10 years (*n* = 7,097)**				
Age (continuous variable)	0.16	0.01	1.17 (1.14–1.20)	<0.001
Gender (female)	−0.06	0.06	0.94 (0.84–1.06)	0.310
Patient type (inpatients)	1.39	0.17	4.00 (2.89–5.54)	<0.001
Diagnosis type (URTI)	0.33	0.09	1.39 (1.16–1.67)	<0.001
**11–27 years (*n* = 763)**				
Age (continuous variable)	−0.08	0.03	0.92 (0.87–0.98)	0.005
Gender (female)	0.13	0.17	1.14 (0.82–1.58)	0.440
Patient type (inpatients)	1.70	0.61	5.47 (1.66–18.02)	0.005
Diagnosis type (URTI)	0.36	0.22	1.43 (0.93–2.18)	0.101
**28–41 years (*n* = 368)**				
Age (continuous variable)	0.01	0.04	1.01 (0.94–1.09)	0.827
Gender (female)	0.14	0.29	1.15 (0.65–2.02)	0.633
Patient type (inpatients)	0.19	1.09	1.21 (0.14–10.30)	0.860
Diagnosis type (URTI)	0.29	0.29	1.34 (0.77–2.35)	0.306
**>41 years (*n* = 332)**				
Age (continuous variable)	−0.02	0.02	0.98 (0.95–1.01)	0.209
Gender (female)	−0.31	0.34	0.74 (0.38–1.43)	0.367
Patient type (inpatients)	2.08	0.76	8.04 (1.83–35.27)	0.006
Diagnosis type (URTI)	0.37	0.33	1.44 (0.76–2.75)	0.265

a The odds ratios (ORs) for each variable were adjusted for the other three variables included in the logistic regression analysis. Female, URTI, and inpatients were used as the reference groups. URTI, upper respiratory tract infection.

### 3.5 Weekly changes in pertussis positivity rates during an upsurge

**Figure 3:**
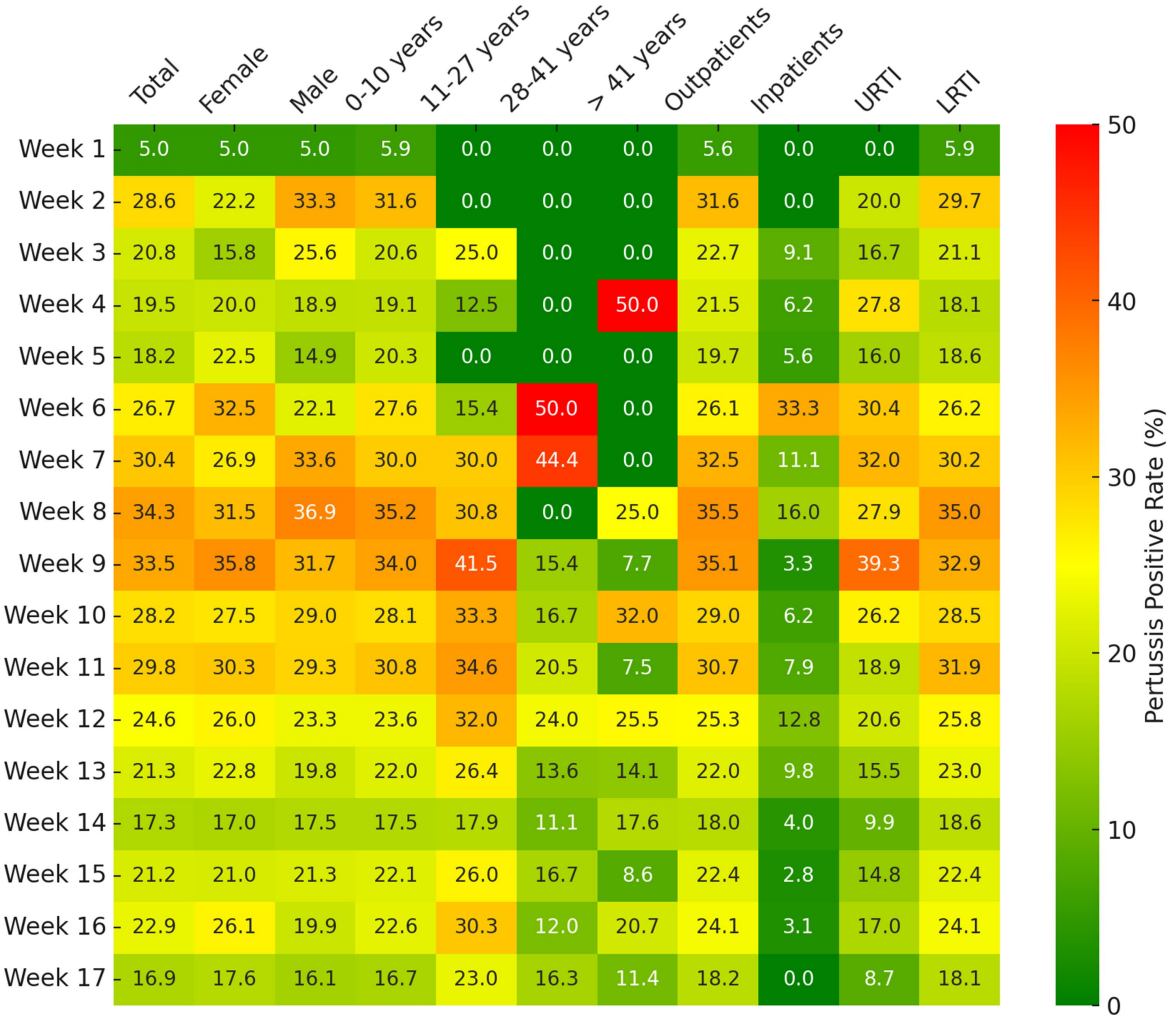
Weekly pertussis positive rates among different population groups following an upsurge (February, 2024 to May, 2024). URTI, upper respiratory tract infections; LRTI, lower respiratory tract infections.

Figure 3 displayed the weekly changes in pertussis positivity rates among different population groups during a rapid upsurge among patients with ARTI from February to May 2024. Overall, the pertussis positivity rate maintained a high level, around 30%, from week 7 to week 11 (March 11 to April 14). Particularly, in week 8 (March 18 to March 24), the positivity rate peaked at 34.29%, and then gradually declined, reaching 16.92% by late May. From February to May, the majority of pertussis cases occurred in the 0–10 and 11–27 age groups, with positivity rates of 25.36 and 28.57%, respectively; the weekly pertussis positivity rates for these two age groups were consistently higher than those in the 28–41 (17.04%) and over 40 (16.31%) age groups, with statistical significance (all *p* < 0.001). Additionally, patients with LRTI generally showed higher pertussis positivity rates compared to those with URTI, and outpatient cases also exhibited higher rates than inpatient cases.

## 4 Discussion

This study systematically analyzed the pertussis positivity rates among 8,560 patients with ARTI treated in three hospitals in Zhejiang Province from January 2023 to May 2024, revealing the epidemiological dynamics and characteristics of pertussis in the post-COVID-19 era. The results showed that pertussis positivity rates exhibited fluctuating growth during the study period, particularly peaking in early 2024 before subsequently declining. Age, and diagnosis type were major factors influencing positivity rates, with notably higher rates observed among children and adolescents aged 5 to 17, potentially due to their higher frequency of interactions in community and school settings. Additionally, the study found that the pertussis positivity rates for patients with LRTI and outpatients were significantly higher than those for URTI and inpatients. These findings may provide important reference information for the prevention and control of pertussis, especially in the formulation and implementation of public health strategies.

Due to variations in immune capabilities, social environments, and social interactions, individuals face different risks of infectious diseases at various stages of life (Wood et al., 2021). For instance, infants are highly susceptible due to their still-developing immune systems (Semmes et al., 2021). Due to frequent interactions in school and other social settings, children and adolescents aged 4–18 represent a population group with specific risks of infection (Read et al., 2021). Young adults are likely exposed to a broader range of pathogens due to extensive social and occupational activities (Acke et al., 2022). Moreover, middle-aged to older adults may have increased susceptibility due to immune senescence and chronic health conditions (Saavedra et al., 2023). In this study, due to the average protective duration of the DTaP vaccine lasting about 3–4 years, and with only 10% of children retaining protection 8 years after their last vaccine dose (McGirr and Fisman, 2015; Domenech de Cellès et al., 2019), we have created a separate age group for those aged 4–8 years (5–10 years old) post the final vaccine dose. At the initial stage of our analysis, the population was divided into age groups of 0–4 years, 5–10 years, 11–18 years, 19–40 years, and over 40 years. In our findings, although the 5–10 years age group was within the vaccine protection period compared to older age groups, this group exhibited the highest pertussis positivity rate. This was likely due to their relatively weaker immune systems, coupled with their frequent interactions within schools and communities. These environments are typically characterized by dense populations and frequent social activities, which accelerate the transmission of respiratory diseases (Sabin et al., 2020). Conversely, the pertussis positivity rate among the infants was relatively low. Although children in this age group have an underdeveloped immune system making them theoretically more susceptible (Borghesi et al., 2020), the protective environment provided by families and their limited social contacts substantially reduce their risk of infection.

After a detailed analysis using the GAM to explore the relationship between age and pertussis infection rates, we identified a wave-shaped non-linear relationship, indicating varying risks of infection across different age groups. These findings once again highlighted significant differences in pertussis infection risk at certain life stages. Notably, peaks in early childhood and mid-adulthood were associated with diminished vaccine efficacy or changes in exposure to pertussis. This model’s ability to capture these subtle variations underscored the importance of implementing age-specific strategies in the prevention and control of pertussis. However, between 2018 and 2022, the incidence of pertussis in China ranged from 0.32 to 2.71 per 100,000 individuals, with infants under 1 year old accounting for 52.40%, children aged 5 to 9 years old accounting for 13.01%, and children and adults aged 10 years and older comprising only 2.49% (Mei et al., 2024). It is important to emphasize that the pertussis infection rates among children and adults are substantially lower than those among infants, a finding inconsistent with our research. This discrepancy may be attributed to two potential factors: (1) The gradual relaxation of COVID-19 control measures in China in December 2022, which could have altered the transmission dynamics of infection (Ryu et al., 2021; Yi et al., 2023). (2) Previously, the incidence rates among adolescents and adults may have been significantly underestimated due to atypical symptoms (Skoff et al., 2015; Yao et al., 2022).

The pertussis positivity rate among patients with LRTI was significantly higher compared to those with URTI in the present study. This finding aligns with studies on other pathogens, which often show higher detection rates of certain bacteria or viruses in LRTI (Dung et al., 2024). For instance, pathogens such as *Streptococcus pneumoniae* and *Haemophilus influenzae* are more frequently detected in cases of lower respiratory tract infections (Abdeldaim et al., 2010; Wang et al., 2021). This might have been because the lower respiratory tract provided a more suitable environment for the growth and proliferation of these pathogens. Particularly, secondary pathogens may have found it easier to invade following a primary viral (or other bacterial) infection, which could have weakened the host’s immune defenses and further increased susceptibility to other infections. This pattern of infection highlighted the complex interactions between different pathogens and the host’s immune response, emphasizing the importance of considering both primary and secondary infections in the management of respiratory diseases. Additionally, in the lower respiratory tract, increased fluid accumulation and damage to the mucosal surface may have facilitated the adherence and proliferation of these pathogens (Hall-Stoodley and McCoy, 2022; Pérez-Cobas et al., 2022). In contrast, URTI often manifest with milder symptoms similar to the common cold, which might not routinely warrant diagnostic testing for pathogens (Krantz et al., 2020; Qavi et al., 2020). This difference in testing frequency could also explain the higher pertussis positivity rates observed in patients with LRTI in our study. These observations underscore the importance of considering the site of infection in the management of respiratory infections and the necessity of employing distinct strategies for different types of respiratory infections in public health practices.

Additionally, it is noteworthy that in this study, the positivity rate of pertussis among hospitalized patients was significantly lower than that of outpatients. This phenomenon could be related to the severity of conditions in hospitalized patients. Typically, respiratory infections requiring hospitalization present with more severe symptoms and are often caused by more lethal respiratory pathogens such as *Streptococcus pneumoniae* (Sender et al., 2021), rather than pertussis. Pertussis often exhibits relatively mild initial symptoms, leading patients with less severe manifestations to seek outpatient rather than inpatient care. Another possible explanation is that hospitalized patients are subject to more stringent monitoring and infection control measures within the hospital setting; hospitals generally implement more detailed isolation and disinfection procedures for inpatients, reducing the risk of cross-infection (Mazzeffi et al., 2021), which may decrease the transmission of pertussis among hospitalized patients.

This study has several limitations. First, this study indeed lacked specific data on the pertussis vaccination coverage rates of the subjects, as well as information on their pertussis vaccination schedules and the specific times when their protective antibody levels began to decline. This resulted in the study not sufficiently emphasizing the critical period for additional vaccination. Second, this study utilized opportunistic sampling rather than systematic active surveillance, which may impact the representativeness and accuracy of the findings. Furthermore, this study included only data on pertussis positivity among patients with ARTI who sought medical care, not the general population. Lacking specific data on the number of positive cases per 100,000 inhabitants, it did not allow for quantification of the overall incidence rate, thus limiting the ability to fully demonstrate the impact of the disease. Third, this study did not include data on pertussis infections prior to and during the COVID-19 pandemic, and therefore was unable to provide a comprehensive analysis of the changes in pertussis cases before, during, and after the pandemic. This may affect our understanding of the overall trends in pertussis. Forth, the study was conducted in only three hospitals in Zhejiang Province, which may not represent the entire region or other areas, limiting the generalizability and accuracy of the findings; furthermore, the lack of specific information about the catchment areas of these hospitals limits our ability to fully evaluate the representativeness and scope of the study data. Additionally, although we had adjusted for several confounding factors, there may still be uncontrolled variables such as specific social behaviors and personal hygiene practices of the patients, which could influence the transmission and positivity rate of pertussis. Future research should include more diverse populations, cover broader geographical areas to provide a more comprehensive understanding of the epidemiology of pertussis post-COVID-19.

## 5 Conclusion

This study demonstrated that post-COVID-19, the positivity rate for pertussis peaked in early 2024 and subsequently declined, with children and adolescents aged 5 to 17 showing higher rates than other age groups, differing from the infection rate distributions observed before and during the pandemic in China. This change was likely linked to the stringent non-pharmaceutical interventions implemented during the pandemic and gradually relaxed thereafter. It suggested that although children are vaccinated against pertussis at birth, booster vaccinations should be considered in preschool and adolescence to maintain community immunity. Future research should further explore the potential health benefits of COVID-19 prevention measures on the transmission of pertussis to enhance prevention and control strategies for pertussis.

## Data availability statement

The raw data supporting the conclusions of this article will be made available by the authors, without undue reservation.

## Ethics statement

The studies involving humans were approved by the Ethics Committee of the Jinyi Medical Group, Affiliated Jinhua Hospital, Zhejiang University School of Medicine, with the approval code: (2024) Ethics No. (101). The studies were conducted in accordance with the local legislation and institutional requirements. Written informed consent for participation was not required from the participants or the participants’ legal guardians/next of kin because according to the regulations of the Ethics Committee, the consent for participation is not necessary for this retrospective cross-sectional study.

## Author contributions

HW: Conceptualization, Formal analysis, Writing – original draft. MF: Data curation, Methodology, Writing – review & editing. WC: Formal analysis, Visualization, Writing – original draft. YM: Conceptualization, Writing – review & editing.
